# Streptococcus: An organism causing diseases beyond neglect

**DOI:** 10.1371/journal.pntd.0008095

**Published:** 2020-05-21

**Authors:** Michael F. Good

**Affiliations:** Institute for Glycomics, Griffith University, Gold Coast, Australia; Yale School of Public Health, UNITED STATES

## Introduction

The neglected tropical diseases (NTDs) are a heterogeneous group of diseases that affect some of the poorest people on the planet. They occur predominantly in low- and middle-income tropical countries (LMIC) and amongst indigenous and very poor people living in developed countries. The concept of NTDs was developed by Peter Hotez and colleagues to draw attention to a large group of diseases that carry significant morbidity but were largely ignored when the Millennium Development Goals were established [1]. The NTDs are now recognized by WHO, and the organization has set targets for their control and elimination.

Different organizations have slightly different lists of diseases that make up the NTDs, but approximately 20 diseases are considered NTDs. All but one (snake bite envenomation) are of infectious origin, and some are transmitted by vectors. While NTDs must obviously be neglected and tropical, the Strategic and Technical Advisory Group committee of WHO also mandated that for diseases to be classified as NTDs, they must disproportionately affect people living in poverty and must be amenable to control or elimination (Table 1). The magnitude of the disease burden, in terms of mortality or morbidity, was not a consideration for inclusion or exclusion. However, the burden of individual NTDs is not large in comparison to diseases such as pneumonia, HIV/AIDS, tuberculosis, malaria, and diarrhoeal diseases, for which the annual disability-adjusted life years (DALYs) each exceed 40 million years [2]. Nevertheless, the societal burden of NTDs is extensive as the total annual burden of life lost is estimated to be in excess of 25 million years [3].

**Table 1:** WHO criteria for inclusion as an NTD.

WHO criteria for inclusion as an NTD^1^
1. Disproportionately affect populations living in poverty and cause important morbidity and mortality—including stigma and discrimination
2. Primarily affect populations living in tropical and subtropical areas
3. Are immediately amenable to broad control, elimination, or eradication
4. Are relatively neglected by research

^1^https://www.who.int/neglected_diseases/diseases/Adoption_additional_NTDs.pdf

NTD, neglected tropical disease

### Strep A

Strep A (*Streptococcus pyogenes*, group A streptococcus) causes multiple diseases, including the very common conditions of tonsillitis and pyoderma (with over 700 million incident cases each year). These two conditions are rarely life threatening nor a cause of major morbidity. Tonsillitis and pyoderma could not be considered as NTDs. However, if untreated, very serious illness can follow. Rheumatic fever (RF) will occur in a few percent of untreated patients. This condition exhibits multiple pathologies affecting the heart, brain, joints, and skin, with the most serious being an irreversible autoimmune-mediated scarring of the heart’s valves—a condition known as rheumatic heart disease (RHD)—which can lead to heart failure and embolic strokes. The number of DALYs lost to RHD was recently estimated to exceed 10,000,000, with the highest rates being in Oceania, South Asia, and Africa [4]. Some indigenous populations in Australia, New Zealand, Canada, and the United States suffer extremely high rates of RF (up to 380 per 100,000) [5–8].

Invasive streptococcal disease (ISD) can follow seemingly mild Strep A infections. It can also develop very rapidly with no apparent evidence of antecedent tonsillitis or pyoderma. This can have devastating outcomes as it can quickly lead to necrotising fasciitis and toxic shock, with mortality rates reported between 20% and 50%. The reported incidence rates of ISD are increasing significantly in developed nations such as Canada and New Zealand [9, 10]. Homelessness and other measures of disadvantage are significant risk factors [9–11]. Within developed countries, indigenous populations suffer some of the highest reported rates of ISD. Similarly high rates of ISD are reported amongst the peoples of developing nations and particularly amongst their very young, their elderly, and their women during the puerperium (rates of up to 75 per 100,000 per year) [12]. These numbers are likely to be an underestimate. Rates for DALYs lost due to ISD are not available.

Globally, the total number of deaths attributed to diseases caused by Strep A (principally RHD and ISD) is estimated to exceed 500,000 per annum [13], with the greatest impact being in LMICs.

### Serious streptococcal diseases occur commonly in the tropics, but their distribution co-locates primarily with poverty (addressing WHO criteria #1 and #2 [Table 1])

Streptococcal diseases are common in the tropics but are not restricted to these regions of the world. This applies to the less serious superficial infections, to the serious poststreptococcal sequelae (RHD, poststreptococcal glomerulonephritis), and to ISD. However, tropical countries bear the greatest brunt of pathology. In an article published in 2012, Steer and colleagues [12] reviewed the rates of ISD in industrialized nations (temperate climates), developing nations (tropical climates), and disadvantaged communities from industrialized countries. The rates ranged from 11.6 to 96 per 100,000 for the tropical countries and from 1.6 to 3.8 per 100,000 for the industrialized nations from temperate climates. The rates for disadvantaged communities from industrialized countries (46 to 82.5 per 100,000) were similar to those from developing countries. These reported rates may be an underestimate of the true incidence. The tropical bias for serious streptococcal diseases is due in large part to the fact that many impoverished nations are located in the tropics. Thus, like the accredited NTDs, Strep A diseases are not restricted to tropical countries but closely follow poverty. In his book, *Blue Marble Health* [14], Hotez makes the point that at least 12 million US citizens live with at least one NTD. These citizens are amongst the most impoverished in the US. In Australia, it is the Aboriginal and Torres Strait islander populations, particularly those living remotely in the tropical north and who are also amongst the most impoverished in their country, that suffer the highest rates of RHD. The same applies to ISD, which occurs in all socioeconomic sectors but is most prevalent amongst Aboriginal Australians in the north [15]. In Canada and New Zealand (both non-tropical countries), ISD and RHD are also most prevalent amongst indigenous populations, who represent the most marginalized citizens in their countries [10, 16].

### Strep A diseases are amenable to control (addressing WHO Criterion #3 [Table 1])

RHD was controlled in many developed countries as a result of economic development that provided improved housing, access to primary healthcare facilities, better education, and clean water. However, state-of-the-art medical technology played the critical role. Although the prevalence of RHD was declining from the start of the previous century, it was the discovery of penicillin that could be used to treat tonsillitis that sharply drove down the rate of new cases and provided secondary prophylaxis against recrudescent RHD. The appropriate use of antibiotics for RHD may be in question as rates of RHD remain high in LMICs in spite of some reports of antibiotic overuse [17]. Nevertheless, it is established that appropriate penicillin use combined with accurate laboratory-supported diagnosis and first-rate public health can control RHD and may even eliminate it. As such, serious Strep A disease fulfils this WHO criterion. However, until countries meet target 3.8 of the Sustainable Development Goals and achieve universal health coverage for all their citizens, it is widely accepted that a vaccine will be required for further significant gains towards elimination of RHD. Similarly, a vaccine will be required to prevent ISD, a disease with a high mortality even when treated with aggressive antimicrobial chemotherapy.

### Strep A diseases are neglected by research (addressing WHO Criterion # 4 [Table 1])

There is no definition as to how low funding and research activity must be for a disease to be classified as neglected, and it is difficult to determine the amount of global research funding that is applied to streptococcus. However, public data are available for the world’s largest funder of research, the US National Institutes of Health (NIH). Their website provides a breakdown of NIH funding for various disease categories for which in excess of US$500,000 is spent per annum [18]. In a table listing 288 different conditions for fiscal years 2015 to 2018, streptococcal research is not listed, indicating that less than US$500,000 was invested in Strep A research by the NIH for each of the last 4 years. By comparison, funding for malaria in 2018 was US$202 million, funding for tuberculosis was US$347 million and funding for HIV/AIDS research was US$3 billion. It is possible that some funding for Strep A could fall under “stroke” or “vaccine-related,” but if the amount were significant, then “Strep A,” (or “group A streptococcus” or “*S*. *pyogenes*” or “RF” or “RHD”) would be listed. Remarkably, not a single NTD (Table 2) is listed in the NIH Table. From the perspective of NIH priorities, Strep A is in good company with the NTDs.

**Table 2:** Number of active clinical trials for various disease categories^1^.

Disease	Number of active trials
The ‘Big three’	
Malaria HIV/AIDS Tuberculosiss	116 884 194
NTDs^2^	
Buruli ulcer Chagas disease Dengue Dracunculiasis Echinococcus Foodborne trematodiases African trypanosomiasis Leishmaniasis Leprosy Lymphatic filariasis Deep mycoses Onchocerciasis Rabies Scabies Schistosomiasis Soil-transmitted helminths Snake bite envenomation Taeniasis Trachoma Yaws	3 12 21 0 3 2 6 29 8 4 1 8 12 3 8 5 3 8 6 1
Strep A^3^	3

^1^The number of trials from all countries categorized as “recruiting and not yet recruiting” on the ClinicalTrials.gov website, as of October 2019.

^2^NTDs as defined by WHO and the US Centers for Disease Control and Prevention. *PLOS Neglected Tropical Diseases* has a larger list (available on its website), but it also does not include Strep A.

^3^At the WHO IVR Consultation on group A streptococcal vaccine R&D, 16–17th May, 2018, Wellcome Trust, London, it was proposed, without objection, that “Strep A,” not “GAS,” would be used as the preferred acronym for group A streptococcus.

GAS, group A streptococcus; IVR, Initiative for Vaccine Research; NTD, neglected tropical disease; R&D, research and development

Some data are also available for smaller national funding agencies. The National Health and Medical Research Council of Australia (NHMRC) has provided funding data over the same period of time for Strep A and/or RHD and the “big three” (malaria, tuberculosis, and HIV/AIDS). Funding for Strep A research in 2018 was AU$7.3 million, funding for malaria research was AU$19.6 million, funding for tuberculosis research was AU$5.5 million, and funding for HIV/AIDS research was AU$16 million. It is pleasing to see that NHMRC funding for Strep A has progressively increased from AU$1.3 million in 2009. Two recent initiatives have seen AU$38 million of funding set aside for streptococcal vaccine development. Recognizing the high burden of RHD amongst indigenous citizens of New Zealand and Australia, the Coalition to Advance New Vaccines Against Group A Streptococcus (CANVAS) was established with a AU$3 million grant from the Governments of New Zealand and Australia to bring prospective vaccine candidates forward for clinical trial [19]. More recently, the Medical Research Future Fund announced that a further AU$35 million would be invested to develop vaccine candidates through to phase II trials [20]. While these funds are distributed over several years, they are a very welcome, albeit small, addition to the global research fund. Much more is needed.

While total global funding coming into streptococcal research from all sources (governments, international organizations, industry, and philanthropy) is not known, a “catch all” surrogate that can be used to ascertain the relative degree of support for research on a particular disease is the number of active clinical trials underway. The ClinicalTrials.gov website lists the various clinical trials on its database. The Food and Drug Administration Amendments Act of 2007 requires that all applicable clinical trials (which excludes phase I studies) be submitted to the ClinicalTrials.gov databank. The data are readily accessible and as such provide a surrogate for all research activity that may lead to a product registrable in the US. Table 2 lists the number of trials for: (1) the “big three” diseases (malaria, tuberculosis, and HIV/AIDS); (2) the diseases listed by WHO and the US Centers for Disease Control and Prevention(CDC) as NTDs; and (3) Strep A. While there are several hundred registered active trials for malaria (116), tuberculosis (194), and HIV/AIDS (884), there are three active trials for Strep A—very similar to the number of active trials for the accredited NTDs (e.g., there are eight for leprosy). While the ClinicalTrials.gov website cannot be used to determine the absolute number of research trials worldwide, it is not unreasonable to infer that the amount of global activity in Strep A clinical trial research is more akin to what is occurring in NTD clinical trials than to what is occurring in trials for malaria and HIV/AIDS.

Thus, while there may be no definition for “neglected” in terms of research, it is clear from funding and clinical trial activity that Strep A research is supported far less than research in other diseases with a similar geographical footprint and with a similar level of global disease burden. The level of research activity is similar to the level of activity for the accredited NTDs.

### The importance for listing Strep A as an NTD

When NTDs were first formally discussed [1], their importance alongside the “big three” (HIV/AIDS, tuberculosis and malaria) was emphasized, and it was argued that the NTDs may pose an equal threat to the health of the poor. Furthermore, because their geographical distribution overlapped, success in the fight against HIV/AIDS, tuberculosis, and malaria would require control of the NTDs. Hotez and Aksoy recently reviewed 10 years of progress in NTD control until 2017 and noted tremendous gains with six NTDs progressing towards elimination [21]. It is noteworthy that in the same time frame, there have been significant reductions in the incidence rates for HIV/AIDS, tuberculosis, and malaria. These achievements are likely to be causally linked because comorbidities are a major factor contributing to mortality. Of added benefit, increased public health commitment for one disease results in enhanced availability of treatment options for many other diseases.

Listing serious Strep A disease as an NTD will bring it under the umbrella of organizations whose mission is to encourage investment from all sectors to control and eliminate these diseases. For example, the Global Network for NTDs (an initiative of the Sabin Vaccine Institute), with WHO, aims to raise awareness and funding to control and eliminate NTDs. These initiatives have led to pharmaceutical companies (including Merck, MedPharm, GlaxoSmithKline, Johnson & Johnson, and Pfizer) donating drugs worth in excess of US$1 billion to control a number of NTDs.

Priority activities for a vaccine development roadmap have been discussed [22], and a number of Strep A vaccines are in development [23, 24]; however, the involvement of industry will be critical. Industry has already taken tentative steps towards supporting vaccine development with Merck [25] and Novartis [26] both “putting their toes in the water,” but now a full commitment from industry is required. The Bill & Melinda Gates Foundation have, to date, resisted attempts to fund Strep A vaccine development.

Serious Strep A diseases fulfils all the WHO criteria for listing as an NTD. Their formal inclusion will enhance political, organizational, and societal pressure and will hasten the commitment of industry and lead to the ultimate elimination of these diseases.
